# Diversity and biological activities of endophytic fungi associated with *Catharanthus roseus*

**DOI:** 10.1186/s12866-019-1386-x

**Published:** 2019-01-21

**Authors:** Geethanjali Dhayanithy, Kamalraj Subban, Jayabaskaran Chelliah

**Affiliations:** FA-06, Department of Biochemistry, Indian Institute of Science, Bangalore, Karnataka 560 012 India

**Keywords:** Endophytic fungus, *Catharanthus roseus*, Cytotoxic activity, Apoptotic activity, *Chaetomium nigricolor*, Cancer, Antioxidant potential

## Abstract

**Background:**

The present study involves diversity and bioactivity of the endophytic fungal community from *Catharanthus roseus* inhabiting the coastal region. This study has been conducted hypothesizing that the microbial communities in the coastal regions would tolerate a range of abiotic stress such as salinity, humidity, temperature and soil composition, and it may produce new metabolites, which may possess bioactive property. Therefore in the current study, the cytotoxicity and free radical scavenging potential of the fungal organic extracts have been investigated. Moreover, the apoptotic and the antioxidant potential of the fungus that exhibited the best activity in preliminary screening has also been demonstrated.

**Results:**

Twenty endophytic fungal isolates were obtained from different parts of the plant, and identified using internal transcribed spacer region analysis. Based on the colonization frequency, the dominant genera were found to be *Colletotrichum, Alternaria* and *Chaetomium* with colonization frequency % of 8.66, 7.00 and 6.33, respectively*.* It was observed that the species diversity and richness was the highest in bark followed by leaf and stem regions of the plant. On screening the fungal ethyl acetate extracts for cytotoxicity against the HeLa cells, the *Chaetomium nigricolor* extract exhibited potent cytotoxic activity of 92.20% at 100 μg mL^− 1^ concentration. Comparison between the different organic extracts (ethyl acetate, chloroform, dichloromethane and hexane) of *Chaetomium nigricolor* mycelial and culture filtrate, it was observed that the mycelial as well the culture filtrate ethyl acetate extracts and the culture filtrate hexane extract showed significant cytotoxic potential against the HeLa and MCF-7 cells, respectively. The apoptotic- and mitochondrial membrane depolarisation-induction potential of the *Chaetomium nigricolor* ethyl acetate extract has also been demonstrated in this study. Further the screening of antioxidant potential of the ethyl acetate fungal extracts using DPPH scavenging assay showed that *Chaetomium nigricolor* extract exhibited potential activity with a significant EC_50_ value of 22 μg mL^− 1^. The ethyl acetate extract of *Chaetomium nigricolor* also exhibited superoxide radical scavenging potential.

**Conclusion:**

These results indicated that diverse endophytic fungal population inhabits *Catharanthus roseus*. One of the fungal isolate *Chaetomium nigricolor* exhibited significant cytotoxic, apoptotic and antioxidant potential.

**Electronic supplementary material:**

The online version of this article (10.1186/s12866-019-1386-x) contains supplementary material, which is available to authorized users.

## Background

Endophytes are microorganisms that generally reside within plants without causing any disease symptom [1]. These microorganisms interact with the host plant, and in turn, the plants to some extent modulate the metabolic process of these endophytes to produce molecules that could manifest protective functions towards the microbe and the host [2]. Endophytic microorganisms also exhibit interspecies interaction via chemical signals [2] and these signaling molecules may at times becomes essential for host’s survival fitness. Recently, endophytic fungi have gained impetus due to their enormous potential to produce a myriad of medicinally important metabolites [3–7]. Therefore, exploring endophytic fungi that inhabit plant species with medicinal properties would provide ample opportunities to discover new metabolites with potential bioactivity [8–10].

*Catharanthus roseus (C. roseus),* a widely prevalent herb is a native of the subtropical area and belongs to the family Apocynaceae. The plant is a producer of several secondary metabolites and so far around 130 alkaloids alone have been identified [11]. The plant is known for its varied medicinal potential ranging from anticancer, antidiabetic, antihypertensive, antibacterial, antifungal and antioxidant effects [12–14]. This plant has been traditionally used in the treatment of fever, rheumatism and fatigueness. The plant also possesses blood coagulation property [11]. Several phenolic compounds of the plant have been noted to exhibit in vitro antioxidant potential [15]. Vincristine and vinblastine are two important indole alkaloids isolated from this plant and are used to treat leukemia and Hodgkin’s lymphoma [16]. Earlier reports have shown that the endophytes that reside inside this plant have the potential to produce host-specific metabolites, vincristine and vinblastine [17–19]. Recently, it has been reported that the organic extracts of endophytic fungi that inhabit *C. roseus* plant showed significant cytotoxicity against the selected cell lines [20]. Moreover, endophytic fungi from *C. roseus* have also been reported to produce few novel compounds with potential cytotoxic activities [21]. The endophytic actinomycetes that inhabit *C. roseus* plant have also been noted for their antioxidant activity [22].

So far, there is only one report on studies involving the diversity of endophytic population from *C. roseus,* where the authors have explored the endophytic community of *C. roseus* inhabiting the northern regions of India [23]. Endophytic fungi are a limitless source for novel metabolites and endophytes from plants growing in special ecological niches could have the ability to produce a myriad of secondary metabolites. It is hypothesized that the endophytic fungi residing within this plant obtained from the high salinity region would possess ability to produce various kinds of bioactive secondary metabolites in order to protect the host-plant from salinity-induced abiotic-stress [24, 25] and these metabolites may possess medicinal properties. Therefore, in this study, we chose to isolate endophytic fungi from *C. roseus* grown in Mahabalipuram, Chennai, which is a geographical location with high salinity, study its diversity and screen them for the production of novel anticancer and antioxidant secondary metabolites.

## Methods

### Composition of liquid and solid media

All media were prepared in double distilled water and autoclaved at 121 °C for 20–30 min depending on the volume. The pH of the media was adjusted to 6.8 ± 2. One liter of each media is composed of the following ingredients along with streptomycin at a concentration of 250 mg L^− 1^.

1) Modified medium-1 broth (M-1D)B: 30 g sucrose, 5 g ammonium tartrate, 0.5 g yeast extract, 1 g soytone, 280 mg Ca_2_ (NO)_3_, 80 mg KNO_3_, 60 mg KCL, 360 mg MgSO_4,_ 20 mg NaH_2_PO_4,_ 1.4 mg H_3_BO_3_, 5 mg MnSO_4_, 2.5 mg ZnSO_4_, 0.7 mg KI; 2) Potato dextrose broth (PDB): 200 g of potato infusion and 20 g dextrose; 3)Sabouraud broth (SDB):40 g dextrose and 10 g peptone; 4) Nutrient broth (NtB): 5.0 g peptone, 2.0 g yeast extract, 5.0 g NaCl. This composition of each media with 1.5% agar served as solid media.

### Collection of plant material and isolation of endophytic fungi from *C. roseus*

Isolation of endophytic fungi from *C. roseus* was carried out using the same protocol as mentioned in a previous report [26]. Twenty leaves, ten stem samples, six root samples and twenty bark samples used for the isolation of endophytic fungi were procured from a *C. roseus* plant found in the coastal areas of Mahabalipuram (12.6269 °N, 80.1927 °E), Tamil Nadu, India. The samples collected were washed with water, and cut into small square pieces of 5 mm. The explants were then washed for 2 mins in each sterilizing solutions viz., 70% ethanol, and 0. 1% sodium hypochlorite and finally with autoclaved double distilled water for 2 mins. Further, the samples were dabbed with sterile filter paper. The surface-sterilized explant fragments were placed in Petri dishes (10 segments in each Petri dish) containing PDA amended with streptomycin at a concentration of 250 mg L^− 1^ to prevent bacterial contamination. Once the endophytic fungi were evident from the edges of the explant the hyphal tips of the newly emerging endophytic fungi were transferred using the forceps to new PDA plates and maintained further.

### Identification, colonization frequency and phylogenetic analysis of the endophytic fungi

The fungal isolates were identified by their morphological characteristics and also by sequencing the internal transcribed spacer (ITS) regions of each isolate. Genomic DNA was isolated using the same protocol as mentioned earlier [27]. PCR amplification of the fungal genomic ITS region 1 and 2 was performed using the following primers: ITS1- Forward primer (TCCGTAGGTGAACCTGCGG) and ITS4- Reverse primer (TCCTCCGCTTATTGATATGC) using same reaction mixture and amplification condition as mentioned earlier [28]. The PCR products were directly sequenced by the Xceleris Corporation using the same primers. The sequences of the ITS regions were compared with the existing species sequences in GenBank using BLASTN software. The sequences and the nomenclature of each species were submitted to GenBank (accession number is shown in Table 1). Colonization frequency (CF) % of each endophytic fungus isolated from *C. roseus* plant was calculated using the formula (Nf / Nt) × 100, where N_f_ is the number of segments colonized by each isolate and Nt is the total number of segments [26].

**Table 1:** Endophytic fungi isolated from different parts of *C. roseus* plant

Isolate number	Isolate accession code no	Closest match	% Identity
1	KY659059	*Lasiodiplodia theobromae*	100
2	KY659064	*Cophinforma mamane*	99
3	KY659061	*Fusarium decemcellulare*	99
4	KY659060	*Colletotrichum capsici*	99
5	–	Unidentified	–
6	–	Unidentified	–
7	KY659069	*Lasiodiplodia theobromae*	99
8	–	Unidentified	–
9	KY659068	*Fusarium solani*	98
10	KY659067	*Lasidiplodia theobromae*	100
11	KY659054	*Chaetomium nigricolor (Also known as Amesia nigricolor)* [46]	99
12	KY659066	*Lasiodiplodia theobromae*	99
13	KY659065	*Lasiodiplodia theobromae*	100
14	KY659058	*Colletotrichum* sp.	99
15	KY659063	*Phoma putamium strain*	99
16	KY659057	*Lasiodiplodia theobromae*	100
17	KY659062	*Lasiodiplodia theobromae*	99
18	KY659056	*Alternaria longipes*	100
19	KY659055	*Colletotrichum*	99
20	–	Unidentified	–

ITS sequences similar to the ITS sequences of isolates used in the study were obtained from GenBank via the BLASTn analysis. Sequences were subjected to multiple sequence alignment by using CLUSTAL software [29] and further the gaps were removed from the sequences. Highly relevant sequences were used for the construction of the phylogenetic tree using maximum parsimony via the MEGA 4 [30] software. *Amanita muscaria* and *Sporendocladia bactrospora* were used as an out group.

### Calculation of fungal diversity

The diversity of endophytic fungi that inhabited *C. roseus* were evaluated using different indices such as Simpson’s dominance (D), Simpson’s diversity index (1- D), Shannon’s diversity index, Evenness, Menhinick’s index (Dmn), Margalef’s richness (Dmg), Equitability ‘J’, Berger Parker Dominance and inverted Berger Parker Dominance [31–33] .

### Growth of endophytic fungi and extraction of secondary metabolites

The endophytic fungi were inoculated in 500 mL flasks containing 200 mL of PDB and incubated in the dark at 30 °C for 21 days. Four agar plugs (9 mm diameter) containing mycelia were used as inoculum. After 21 days, the culture was filtered through 2 layers of cheesecloth to separate mycelia and culture filtrate. The mycelia thus harvested, were dried at 50 °C overnight and their dry weight was determined. The dried mycelia were then powdered using liquid nitrogen and were extracted with a 5X volume of ethyl acetate whereas, the culture filtrate was extracted using 2X volume of ethyl acetate. The organic phase was separated using a separating funnel and evaporated to dryness using a vacuum rotary evaporator at 45 °C. The residue was weighed and dissolved in 0.02% DMSO and used for further cell culture experiments. For antioxidant assays, the residue was dissolved in methanol and used.

### Optimization of solvent for extraction of secondary metabolites

Four (9 mm diameter) agar plugs of *Chaetomium nigricolor* (*C. nigricolor)* was inoculated in 200 mL of M-1 dB medium (growth medium in which *C. nigricolor* produced metabolites with best cytotoxicity-see Additional file 1; Table S1) incubated in the dark at 30 °C under static condition and fermented for 21 days. After 21 days the fermented culture was harvested and extracted using the same protocol as mentioned above, using four different solvents namely ethyl acetate (EA), chloroform, hexane and dichloromethane. The extracts were dried completely and dissolved in 0.02% DMSO for further use in cell culture experiments.

### Human cancer cell lines and maintenance

HeLa (Human cervical adenocarcinoma), MCF-7 (Human breast adenocarcinoma) cell lines and HEK 293 T (non cancerous-human embryonic kidney cells) were purchased from the National Centre for Cell Sciences (NCCS), Pune, India. Both the cells line were grown using Dulbecco’s modified Eagle’s medium (DMEM) supplemented with 10% fetal bovine serum (FBS). The medium was supplemented with penicillin (100 IU mL^− 1^) and streptomycin (100 IU mL^− 1^). The cell lines were maintained in a humidified 5% CO_2_ atmosphere at 37 °C.

### Cytotoxicity assay

Fungal extracts were evaluated for their cytotoxicity by the MTT assay [34]. The cancer cells were maintained in DMEM medium. Approximately 4000 cells/ well were seeded into each well of 96-well plate and left to acclimatize overnight. The cancer cells were then treated with fungal crude extracts (100 μg mL^− 1^ of EA extracts were used for the preliminary screening of all the isolates to evaluate their cytotoxicity and for testing the cytotoxicity of different organic solvent extracts of *C. nigricolor* 5, 10, 15, 25, 50 and 100 μg mL^− 1^ concentrations of the extracts were used) for 24 h. Post-treatment duration, 20 μL of 5 mg mL^− 1^ of MTT solution (prepared in PBS) was added to each well and further incubated for 2 h. 100 μL of DMSO was added to each well to solubilize the purple formazan crystals and optical density (OD) was measured at 595 nm using a microplate reader (Molecular devises, USA). The percentage inhibitions of growth exhibited by the cancer cells on treatment with the crude extracts were evaluated using the formula, growth inhibition (%) = [1-[OD of treated cells/ OD of untreated cells] × 100]. Vincristine (2 μg mL^− 1^) was used as positive control.

### Measurement of mitochondrial membrane potential using JC-1 staining

The change in the mitochondrial membrane potential was determined by JC-1 (5,5′,6,6′-tetrachloro-1,1′,3,3′-tetraethylbenzimidazolcarbocyanine iodide) staining following the procedure as described in an earlier report [35]. HeLa cells (1 X 10^5^ cells) per well were grown per well in a 24 well plate for overnight. The HeLa cells were then treated with 10 μg mL^− 1^, 25 μg mL^− 1^ and 50 μg mL^− 1^ of the EA extract of *C. nigricolor* for 24 h. Post-treatment, 0.2 μM of JC-1 dye was added to the untreated cells and treated cells, incubated in the dark for about 37 °C for 15 mins. The cells were then harvested, washed twice with PBS and analyzed using a FACS instrument (VERSE). The data were plotted using FACS DIVA software. 2,4 DNP was used as positive control.

### Annexin V FITC/PI flow cytometry

Annexin V FITC/ PI flow cytometry was performed to evaluate whether the EA extract of *C. nigricolor* induced apoptosis in HeLa cells using the protocol mentioned by Miller et al [36]. HeLa cells were seeded in a 24-well plate at a density of 1 X 10^5^ cells per well. The cells were allowed to acclimatize overnight, further they were treated with 10 μg mL^− 1^ and 25 μg mL^− 1^ of the EA extract of *C. nigricolor* for 24 h. The cells were subsequently stained with 1 μL of Annexin V-FITC stain, and 0.5 μg mL^− 1^ of propidium iodide (PI) stain. The stained cells were further analyzed using FACS Verse flow cytometer (Becton Dickinson, USA) using excitation (λex)/ emission (λ em) of 488/520 nm for AnnexinV-FITC and 540/630 nm for propidium iodide and analyzed using FACS DIVA software.

### Free radical scavenging assay

The free radical scavenging activity of fungal organic extracts was measured by DPPH assay [37, 38]. Briefly, to 200 μL of 0.1 mM of DPPH solution (prepared by dissolving DPPH in ethanol) different concentrations of EA extracts (0, 10, 25, 50, 100 and 200 μg mL^− 1^) were added, vortexed vigorously and incubated for 30 mins in dark at room temperature. Post incubation, the absorbance was measured at 517 nm using a microplate reader with ethanol as blank. The percentage of radical scavenging potential was calculated using the formula = (1-(Abs_(517 nm)_ of the sample/ Abs_(517 nm)_ of the control)) × 100. Ascorbic acid at a concentration of 5, 15, 25, 50 and 100 μg mL^− 1^ was used as a positive control.

### Superoxide anion scavenging activity

To a mixture of 1 mL of Nitroblue Tetrazolium solution (150 μM NBT) and 1 ml NADH (468 μM in 100 mM PBS), different concentrations of EA extract (0,10, 25, 50, 100 and 200 μg mL^− 1^) of *C. nigricolor* were added, and the reaction was initiated by adding 100 μl of phenazine methosulphate (PMS) solution (60 mM PMS in 100 mM phosphate buffer, pH 7.4) and was further incubated at 25 °C for 5 mins. The absorbance at 560 nm was measured against PBS solution used as a blank. The percentage of superoxide anion radical scavenging potential was calculated using the formula = (1-(Abs_(560 nm)_ of the sample/ Abs_(560 nm)_ of the control)) × 100 [39]. Ascorbic acid at a concentration of 50 μg mL^− 1^ was used as a positive control.

### Hydroxyl radical scavenging assay

To a mixture of 100 μM FeCl_3_, 100 mM EDTA, 3.75 mM 2-deoxyribose, and 1 mM H_2_O_2_ different concentrations (0, 10, 25, 50, 100 and 200 μg mL^− 1^) of *C. nigricolor* EA extract were added. The mixture was incubated at 37 °C for 1 h. Post incubation 1 mL of 2% TCA (Trichloroacetic acid) and 1% TBA (Thiobarbituric acid) were added, and further incubated in a boiling water bath for 15 mins. After cooling, the mixture was centrifuged and the absorbance was measured at 535 nm. The percentage of hydroxyl radical scavenging potential was calculated using the formula = (1-(Abs _(535 nm)_ of the sample/ Abs _(535 nm)_ of the control)) × 100 [38]. Ascorbic acid at a concentration of 50 μg mL^− 1^ was used as a positive control.

### Nitric oxide scavenging assay

To 150 μL of sodium nitroprusside, different concentrations (0, 10, 25, 50, 100 and 200 μg mL^− 1^) of EA extract of *C. nigricolor* were added. This reaction mixture was incubated for 150 mins. Post incubation period, 200 μL of Greiss reagent was added and the absorbance was read at 546 nm. The percentage of nitric oxide scavenging potential was calculated using the formula = (1-(Abs _(546 nm)_ of the sample/ Abs _(546 nm)_ of the control)) × 100 [40]. Ascorbic acid at a concentration of 50 μg mL^− 1^ was used as a positive control.

### Statistical analysis

Data for cytotoxicity and antioxidant assays are presented as the means ± standard deviation (SD) for at least two independent experiments (each performed in triplicates), and the means of treated and untreated were analyzed for statistically significant difference using Student's t Test using Graph Pad Prism 7 software. *P* values were also calculated for the same. Data for measurement of mitochondrial membrane potential using JC-1 staining and annexin V FITC/PI flow cytometry experiments were expressed as mean ± SD of three independent experiments.

## Results

### Identification and colonization frequency of endophytic fungi isolated from *C. roseus*

**Fig. 1:**
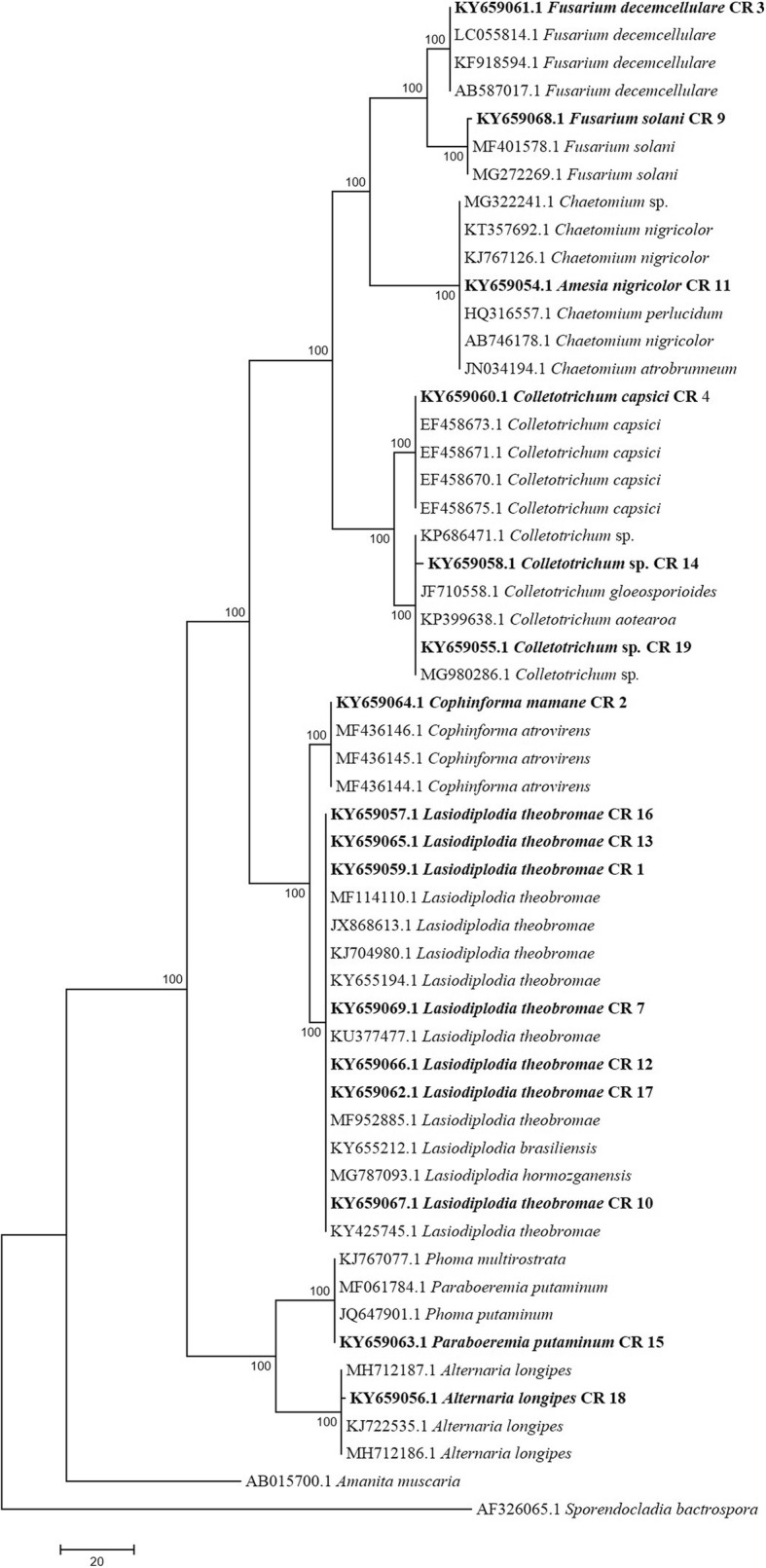
Phylogenetic tree of endophytic fungi isolated from *C. roseus* plant based on ITS region. The phylogenetic tree was constructed using maximum parsimony method. 100% bootstrap value showed each genera were distinguished by monophyletic group in different subclades from outgroup. Nomenclature of the endophytic fungi and the isolate number are indicated

Twenty endophytic fungi were isolated from four tissues (leaf, stem, bark and root) of *C. roseus,* of which 40% of the fungi were from bark, 30% from leaves, 25% from stem and 5% from root. The fungal isolates were identified using morphological features as well as sequence analysis of the ITS regions. The obtained ITS sequences were compared with the sequences in the GenBank repository to identify the fungi. Isolates 5, 6, 8 and 20 have not been identified due to the unavailability of similar sequences in GenBank. The identified fungi with their respective codes, GenBank accession numbers and percentage of identity is given in Table 1. Further, we also constructed a phylogenetic tree using ITS 1 / 5.8S rDNA / ITS 2 sequences using MEGA 4 software. This phylogenetic tree gives the evolutionary relationship between different endophytic fungi under study (Fig. 1).

The colonization frequency of each isolated fungus has been given in Table 2. All isolates identified belonged to a single division, Ascomycota; two classes, namely Dothideomycetes and Sodariomycetes; and seven genera, namely, *Lasiodiplodia, Fusarium, Colletotrichum, Phoma, Cophinforma, Alternaria* and *Chaetomium*. From the colonization frequency it is observed that *Colletotrichum* was the most prevalent genus with a CF % of 8.66, followed by *Alternaria* and *Chaetomium* with a CF% of 7.00 and 6.33, respectively.

**Table 2:** Colonization frequency of different endophytic fungi isolated from different tissues of *C. roseus* plant

Isolate number	Isolate name	Plant organ	CF %
1	*Lasiodiplodia theobromae*	Bark	2.66
2	*Cophinforma mamane*	Leaf	2.00
3	*Fusarium decemcellulare*	Bark	1.33
4	*Colletotrichum capsici*	Bark	7.66
5	Unidentified	Leaf	0.66
6	Unidentified	Bark	1.00
7	*Lasiodiplodia theobromae*	Stem	0.33
8	Unidentified	Leaf	1.00
9	*Fusarium solani*	Bark	2.33
10	*Lasidiplodia theobromae*	Stem	1.33
11	*Chaetomium nigricolor*	Stem	6.33
12	*Lasiodiplodia theobromae*	Stem	3.00
13	*Lasiodiplodia theobromae*	Bark	0.33
14	*Colletotrichum* sp.	Leaf	6.00
15	*Phoma putamium strain*	Bark	0.66
16	*Lasiodiplodia theobromae*	Bark	1.33
17	*Lasiodiplodia theobromae*	Root	2.00
18	*Alternaria longipes*	Leaf	7.00
19	*Colletotrichum*	Leaf	8.66
20	Unidentified	Stem	5.33

CF, colonization frequency

### Fungal diversity

The number of various fungal species from each plant tissues such as bark, stem, leaves and root ranged from 5 to 1. The diversity indices of endophytic fungal species in different plant tissues of *C. roseus* are listed in Table 3. The Shannon and Simpson’s indices were 1.47 and 0.85 for bark, 1.04 and 0.83 for leaves and 0.5623 and 0.5 for the stem, respectively. These diversity indices could not be calculated for endophytes from the root region, because only one fungal species was found in the root region of the plant. In addition, the overall diversity of the endophytic fungal population inhabiting this plant species, represented by Shannon index H′ and Simpson’s index (1-D) were 1.83 and 0.1833, respectively. The species richness of endophytic fungi inhabiting different tissue samples were 1.89, 1.5 and 1 for bark, leaf and stem, respectively. On comparing the evenness value among the bark, stem and leaf, evenness of the species was highest in the leaf and stem, and lowest in the bark. The relative diversity/equitability (J) shows that the leaves had the highest ‘J’ value of 0.9464, followed by bark and stem with a ‘J’ value of 0.9 and 0.81, respectively. The inverted Berger Parker dominance index was highest for the bark, compared to the leaf and stem tissues.

**Table 3:** Diversity of endophytic fungi isolated from different tissues of *C. roseus* plant

Diversity index	Stem	Leaf	Bark
Simpson’s Dominance	0.5	0.16	0.14
Simpson’s (1-D)	0.5	0.83	0.85
Shannon H′	0.5623	1.04	1.47
Eveness	1	0.32	0.28
Meinhenich richness index	1	1.5	1.89
Margeref richness index	0.72	1.44	2.056
Equilability J	0.81	0.9464	0.91
Berger Parker Dominance	0.75	0.5	0.42
Inverter Berger Parker Dominance	1.33	2	2.33

### In vitro cytotoxic activity of endophytic fungal culture extracts against HeLa cell lines by the MTT assay

**Fig. 2:**
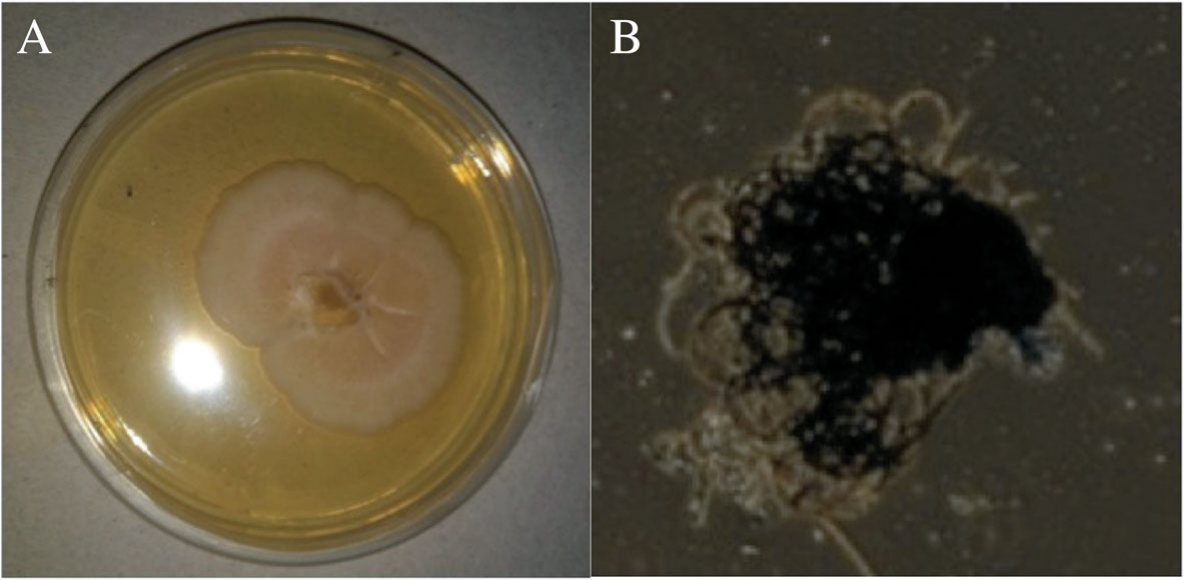
Morphological observation of the fungus *C. nigricolor*. **a**) The colonies after 7 days at 30 °C on PDA plate **b**) Light microscopy image of the *C. nigricolor* spore (40 X magnification)

The ethyl acetate extracts of all the obtained fungal isolates were then assessed for cytotoxic activity against the HeLa cells. The percentage of cytotoxic activity exhibited by EA extracts of all the isolates is given in Table 4. Among the twenty fungal organic extracts screened for cytotoxicity towards the HeLa cell line, extract of isolate 11 (*Chaetomium nigricolor* otherwise called *Amesia nigricolor*) showed a strong cytotoxicity of 92.20%. Since *C. nigricolor* EA extract showed the best cytotoxicity it was chosen for further studies. The EA extract of *C. nigricolor* was also evaluated for its cytotoxicity against the HEK 293 T cells, the data (see Additional file 1; Fig. S1) shows that the EA extract did not exhibit any significant cytotoxicity against it. The culture and spore morphology of *C. nigricolor* is given in Fig. 2.The isolates 6 and 13 also showed moderate cytotoxic activity. The other fungal extracts displayed no appreciable activity against the HeLa cancer cell line.

**Table 4:** Cytotoxicity of the ethyl acetate extracts (concentration-100 μg mL^−1^) of twenty endophytic fungi isolated from *C. rosues* on HeLa cells

Isolate number	Isolate name	Concentration μg mL^− 1^	Percentage growth inhibition on HeLa cells
1	*Lasiodiplodia theobromae*	100	37.10 ± 0.48
2	*Cophinforma mamane*	100	34.69 ± 0.31
3	*Fusarium decemcellulare*	100	9.29 ± 0.45
4	*Colletotrichum capsici*	100	35.22 ± 0.37
5	Unidentified	100	36.18 ± 0.31
6	Unidentified	100	46.72 ± 0.47
7	*Lasiodiplodia theobromae*	100	40.33 ± 1.34
8	Unidentified	100	26.80 ± 0.34
9	*Fusarium solani*	100	40.85 ± 0.60
10	*Lasiodiplodia theobromae*	100	40.33 ± 0.65
11	*Chaetomium nigricolor*	100	92.20 ± 0.42
12	*Lasiodiplodia theobromae*	100	36.29 ± 0.68
13	*Lasiodiplodia theobromae*	100	52.12 ± 1.2
14	*Colletotrichum* sp.	100	35.59 ± 0.85
15	*Phoma putamium strain*	100	27.04 ± 1.84
16	*Lasiodiplodia theobromae*	100	20.40 ± 0.71
17	*Lasiodiplodia theobromae*	100	27.05 ± 0.42
18	*Alternaria longipes*	100	38.34 ± 0.64
19	*Colletotrichum*	100	44.65 ± 0.03
20	Unidentified	100	34.84 ± 0.05
+ ve control	Vincristine	2 μM	90 ± 0.2

Cell viability was determined by the MTT assay using human cervical cancer cell line, HeLa. Growth inhibitory values are mean ± SD calculated from results obtained from triplicate of two independent experiments (n = 6)

For the extracts of all isolates, the means were significantly different as compared to the untreated cells at *P* < 0.0001

### Cytotoxic effect of the organic extracts of *C. nigricolor* on HeLa and MCF-7 cells

In order to evaluate whether the liquid culture filtrate or the mycelium of *C. nigricolor* contained the cytotoxic principle, and also to evaluate the most suitable solvent to extract the cytotoxic secondary metabolites, the culture filtrate and mycelia of *C. nigricolor* was extracted using organic solvents with varying polarity viz., dichloromethane, chloroform, ethyl acetate and hexane. The extracts thus obtained were evaluated for cytotoxicity on both HeLa and MCF-7 cells by the MTT assay (Table 5). The culture filtrate and mycelial ethyl acetate extracts showed the best cytotoxic activity on HeLa cells with an IC_50_ value of 50 ± 0.02 and 25 ± 0.4 μg mL^− 1^ respectively, compared to other extracts. Whereas the culture filtrate hexane extract of *C. nigricolor* showed the best antiproliferative activity against MCF-7 cells with an IC_50_ value of 45 ± 0.04 μg mL^− 1^. EA- cuture filtrate, −mycelial extract and hexane culture filtrate extract were also evaluated for cytotoxicity against HEK 293 T cells, and it was observed that none of the extracts did exhibit any cytotoxicity against it (see Additional file 1; Fig. S1). Since both the mycelial and culture filtrate EA extracts of *C. nigricolor* exhibited the significant cytotoxicity, in the current study we have pooled the culture filtrate and mycelial ethyl extract of it and further evaluated for its mitochondrial membrane depolarisation- and apoptosis- inducing ability*.*

**Table 5:** Cytotoxicity IC_50_ values of different organic extracts of *C. nigricolor* on HeLa and MCF-7 cells

Extracts of *C. nigricolor* under study	Cytotoxic activity IC _50_ (μg mL^−1^)	
	HeLa	MCF-7
EA mycelial extract	25 ± 0.4	> 100
EA culture filtrate extract	50 ± 0.02	> 100
Chloroform mycelial extract	> 100	> 100
Chloroform culture filtrate extract	> 100	> 100
Dichloromethane mycelial extract	> 100	> 100
Dichloromethane culture filtrate extract	> 100	> 100
Hexane mycelial extract	> 100	> 100
Hexane culture filtrate extract	> 100	45 ± 0.04
+ ve control	Vincristine	1.2

EA, ethyl acetate

Cell viability was determined by the MTT assay using human cervical cancer cell line, HeLa and human breast cancer cell line, MCF-7. IC_50_ values are mean ± SD calculated from results obtained from triplicate of two independent experiments (n = 6)

### Effect of *C. nigricolor* EA extract on mitochondrial membrane depolarisation

**Fig. 3:**
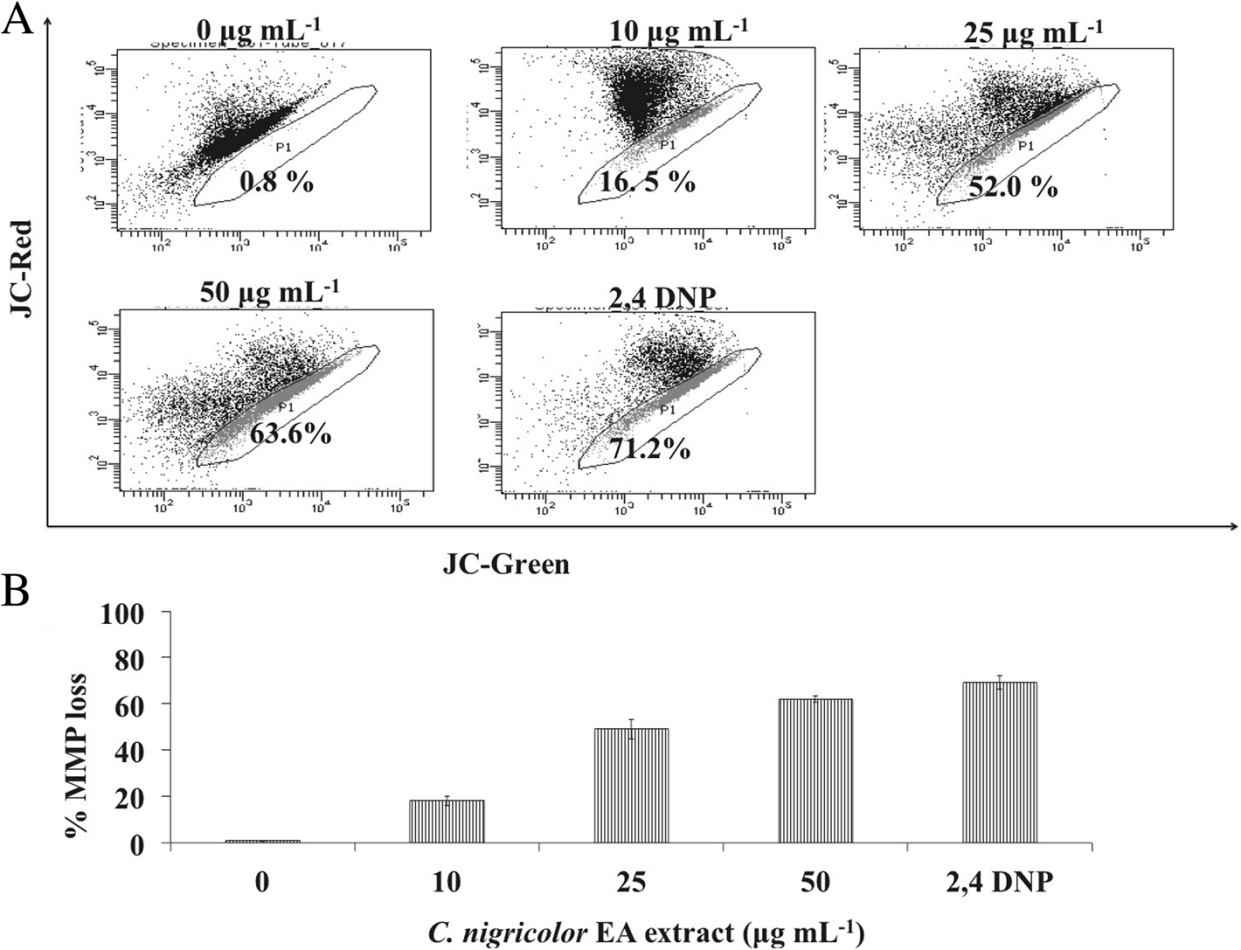
Effect of EA extract of *C. nigricolor* on loss of mitochondrial membrane potential (MMP) in HeLa cells. HeLa cells were treated with the indicated concentrations of the EA extract, stained with JC-1 and subsequently analyzed by flow cytometry, numbers in the bottom right gate represents percentage of cells with low MMP. B) Statistical analysis about loss of MMP obtained in flow cytometer.The experiments were repeated three times and the results are presented as mean ± SD

Mitochondrial membrane depolarization is an early event of apoptosis and can be detected by the JC-1 dye. On treatment with 10, 25 and 50 μg mL^− 1^ of the EA extract of *C. nigricolor* for 24 h, 17.9 ± 2.0%, 49 ± 4.2% and 62 ± 1.41% of mitochondrial membrane potential loss was observed, respectively. This is represented in Fig. 3. 2, 4 DNP was used as a positive control. The result suggests that the *C. nigricolor* EA extracts disrupts the mitochondrial membrane integrity in a concentration dependent manner.

### Effect of *C. nigricolor* EA extract on externalization of phosphatidylserine

**Fig. 4:**
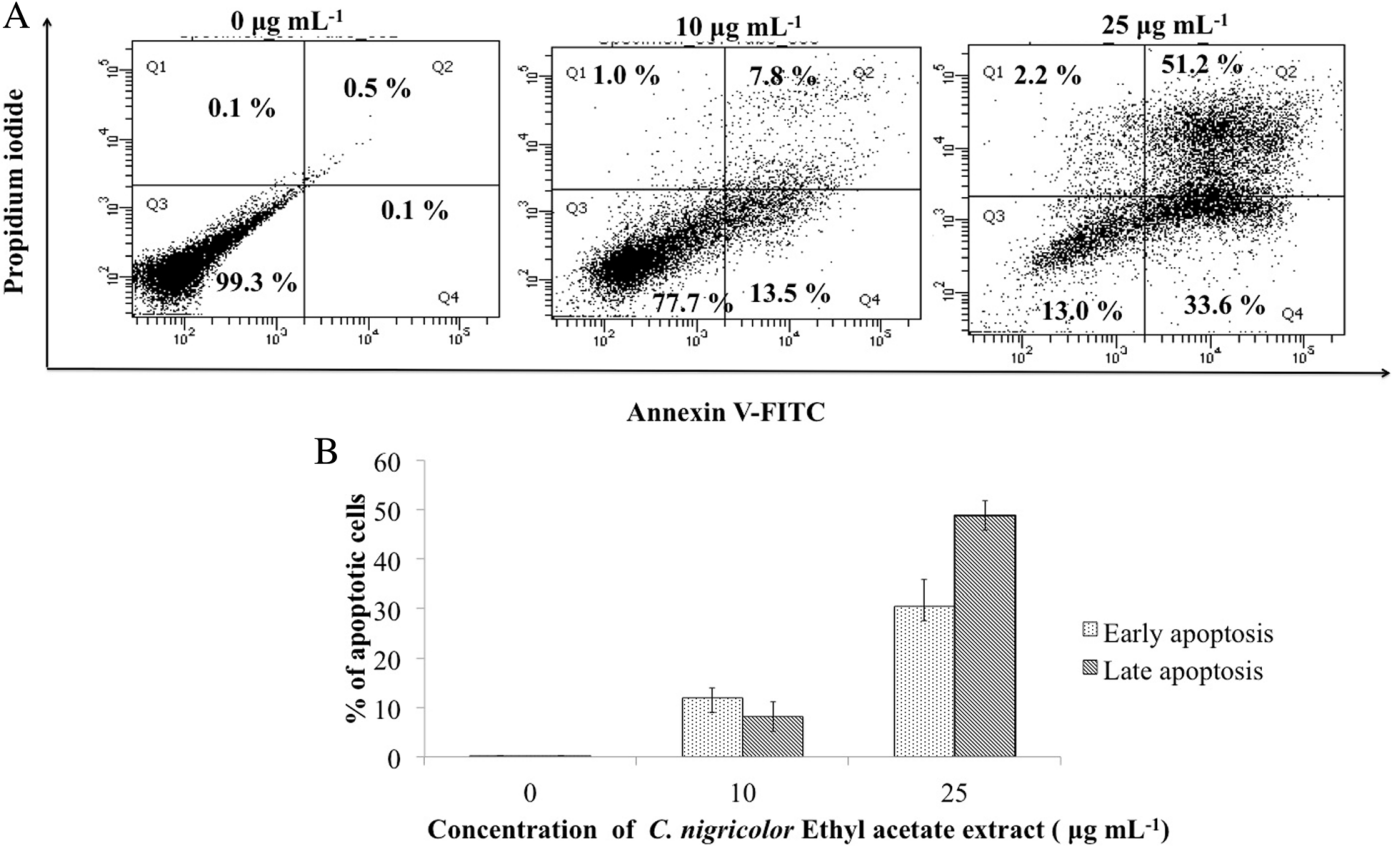
The effect of *C. nigricolor-* EA extract treatment on HeLa cells apoptosis determined by annexin V/ PI double staining. HeLa cells were treated with or without the extract for 24 h. After incubation the cells were double stained with Annexin –V FITC and PI, and analyzed by flow cytometry. Dot-plot represents cell distribution as follows: Lower left (PI – ve/ FITC –ve) living cells, lower right (PI –ve/ FITC +ve) apoptotic cells, upper right (PI +ve /FITC +ve) late apoptotic cells, upper left (PI +ve /FITC -ve) dead necrotic cells. B) Bar diagram representing distribution of early and late apoptotic cells. The data are derived from atleast three independent experiments

Apoptosis induced by the EA extract of *C. nigricolor* in HeLa cells was assessed by flow cytometry analysis using annexin V-FITC /PI double staining. It is well known that the cells in early apoptosis phase are positive for annexin V, whereas cells that are in late apoptosis are positive for both annexin V and PI. Cells that have entered the necrotic phase are positive for PI only. From FACS analysis we were able to infer that the HeLa cells treated with the 10 μg mL^− 1^ of EA extract of *C. nigricolor* for 24 h underwent apoptosis, with 13.9 ± 1.95% of cells in early apoptosis phase and 7.6 ± 2.9% of cells in late apoptosis, and on increasing the concentration to 25 μg mL^− 1^, it was observed that 30.5 ± 5.30% cells were in early apoptosis phase and 48.8 ± 2.96% of the cells were in late apoptosis phase (Fig. 4), indicating the induction of apoptosis in a concentration-dependent manner.

### Free radical scavenging activity of EA extracts of endophytic fungi using DPPH assay

The EA extracts of all the isolated fungi were tested for their free radical scavenging activity using the DPPH assay. Of the 20 fungal extracts that were evaluated for the scavenging activity, *C. nigricolor* EA extract showed significant scavenging activity with an EC_50_ value of 22 μg mL^− 1^. Apart from this extract, the extract of isolate 13 and 19 identified as *Lasiodiplodia theobromae* and *Colletotrichum* also exhibited prominent scavenging activity with an EC_50_ value of 58 ± 0.3 μg mL^− 1^ and 66 ± 0.2 μg mL^− 1^, respectively. Since *C. nigricolor* exhibited the best scavenging activity compared to the other isolates, it was further evaluated for its other antioxidant properties. The list of isolates and their DPPH scavenging potential has been given in Table 6.

**Table 6:** Antioxidant potential of ethyl acetate extract of endophytic fungi isolated from *C. roseus*

Isolate number	Isolate identified as	DPPH scavenging potential EC_50_ (μg mL^− 1^)
1	*Lasiodiplodia theobromae*	> 100
2	*Cophinforma mamane*	> 100
3	*Fusarium decemcellulare*	100 ± 0.01
4	*Colletotrichum capsici*	> 100
5	Unidentified	> 100
6	Unidentified	> 100
7	*Lasiodiplodia theobromae*	> 100
8	Unidentified	> 100
9	*Fusarium solani*	> 100
10	*Lasidiplodia theobromae*	> 100
11	*Chaetomium nigricolor*	22
12	*Lasiodiplodia theobromae*	> 100
13	*Lasiodiplodia theobromae*	58 ± 0.3
14	*Colletotrichum* sp.	> 100
15	*Phoma putamium strain*	> 100
16	*Lasiodiplodia theobromae*	> 100
17	*Lasiodiplodia theobromae*	> 100
18	*Alternaria longipes*	> 100
19	*Colletotrichum*	66 ± 0.2
20	Unidentified	> 100
+ ve control	Ascorbic acid	18 ± 0.2

### Antioxidant activity of EA extracts of *C. nigricolor*

**Fig. 5:**
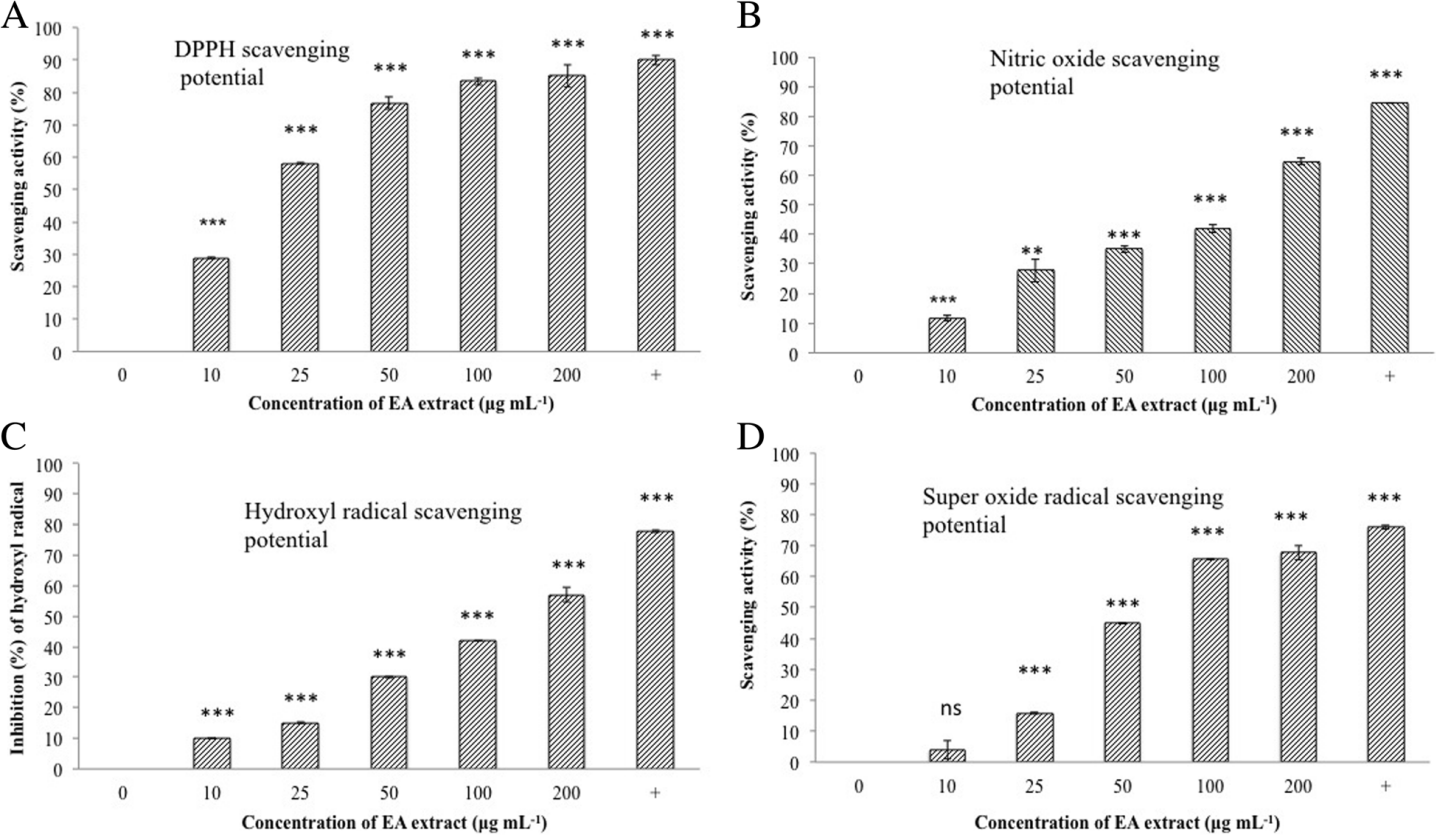
Antioxidant activity of *C. nigricolor*- EA extract **a**) DPPH scavenging activity **b**) Nitric oxide scavenging potential **c**) Hydroxyl radical scavenging activity **d**) Superoxide scavenging activity. Ascorbic acid at a concentration of 50 μg mL^−1^ was used as a positive control for all the antioxidant assays. The data are derived from atleast two independent experiments each performed in triplicates (n = 6). P value was calculated by comparing the means ± SD of the % scavening activity observed with adding and without adding the extract to the reaction mixture, using Student’s T test and the statistical significance are presented as follows in the bar graph: ***,*P* ≤ 0.001; **P ≤ 0.01; ns, *P* > 0.05

The EA extract of *C. nigricolor* was evaluated for its antioxidant properties via its potential to scavenge super oxide, nitric oxide and hydroxyl radicals. The scavenging property of the extract at different concentrations has been represented in the Fig. 5. The EA extract of it exhibited varying degrees of scavenging activity for different radicals. The EA extract of *C. nigricolor* exhibits best DPPH inhibition activity and an average level of superoxide radical scavenging activity with an EC_50_ value of 22 μg mL^− 1^ and 65 μg mL^− 1^, respectively. But the extract was not a good scavenger of hydroxyl radical or nitric oxide radical.

## Discussion

The microbial diversity that the plant harbors coupled with the chemodiversity that the endophytic fungus possesses provides ample opportunity for the discovery of new lead molecules [7]. Apart from the microbial and chemical diversity, the endophyte-host plant interaction also confers the potentiality to the microbes to produce a myriad of novel medicinally useful compounds [2]. Medicinal plants have always been a niche for endophytes and the host –microbial interaction has always instigated the production of several novel metabolite [2] by microbes apart from the compound that are original to the plants (the production of which is facilitated via horizontal gene transfer). Moreover chemodiversity, the traditional use of the plant and region from where the plant is obtained are vital criteria for the isolation of endophytes [41].The need for novel compounds with potential anticancer and antioxidant activity has become inevitable. Therefore in the curent study we have studied the endophytic fungal population from *C. roseus* plant for its diversity, anticancer and antioxidant potential.

Seven different genera of endophytic fungi were isolated from different regions of the plant. In contrary to the previous study conducted by Kharwar et al*,* where most of the fungal species from *C. roseus* belonged to Hyphomycetes [23], in the current study most of the isolated endophytic fungi belonged to Dothideomycetes and Sodariomycetes class. The difference in region from where the plant is isolated could be one possible reason for difference in the isolated microbial community. In the current study, the endophytic fungi that inhabited the plant exhibited moderate diversity and the endophytic fungi were distributed throughout the plant. Though based on the CF%, *Colletotrichum* is the most prevalent species and *Lasiodiplodia theobromanae* was the least prevalent species, *Lasiodiplodia* is one genus which was present in almost all the plant tissues under study.

Colonization frequency data shows that different endophytic fungi inhabited different plant tissues to different extent. This difference in inhabiting potential of the endophytic fungi shows that, each endophyte exhibited different degrees of affinity towards different tissues of the plant. Most of the endophytic fungi isolated in this study were commonly reported endophytes from various medicinal plants.

An activity-based screening procedure was opted in this study, to look out for endophytes with potential bioactivity. The cytotoxic potential of the EA extracts of the isolated endophytic fungi were investigated against human cervical cancer cell line, HeLa. It was observed that the EA extract of the fungus *C. nigricolor* exhibited the best cytotoxicity against the HeLa cell line. Several fungi belonging to *Chaetomium* genus have been noted to produce a myriad of secondary metabolites having bioactive potential. For instance, the fungus belonging to this specific genus was noted to produce exometabolites such as chaetopyranin, chaetoglobosins, sterigmatocystin, chaetracin A and chaeotocin with potential bioactivity [42–45]. The different organic extracts of the fungus *C. nigricolor* were further evaluated for their cytotoxic activity against the HeLa and MCF-7 cells. The EA extract of the fungus exhibited potential cytotoxicity against the HeLa cells, whereas the hexane culture filtrate extract showed significant cytotoxic potential against the MCF-7 cells. It is hypothesized that the ethyl acetate extract has exometabolites that exhibit cytotoxicity against the HeLa cells, whereas the hexane culture filtrate extract could have the exometabolites that exhibit potential cytotoxicity against the breast cancer cell line, MCF-7. Further, in this study, we have also evaluated the mitochondrial membrane depolarisation and apoptosis inducing potential of EA extract of *C. nigricolor,* since this organic extract of the fungus exhibited the best cytotoxic activity and therefore could be used in future for isolation of novel anticancer metabolite(s).

An early event of apoptosis includes mitochondrial membrane depolarization, which in turn leads to disturbance in the respiratory chain and release of cytochrome C into the cytosol. JC-1 dye detects the mitochondrial depolarization, an decrease in the red to green ratio of the dye represents mitochondrial depolarization. The EA extract of *C. nigricolor* induced a mitochondrial membrane depolarization and was scored by FACS analysis. The results also conclusively showed that the EA extract induced apoptosis in HeLa cells via externalization of the phosphatidyl serine, an important hallmark of apoptosis.

Free radicals are known to induce oxidative damage to the body and in turn cause several disorders like cancer, cardiac disorder, cataract, diabetes mellitus, arthritis, and aging. Several research groups have already shown that both medicinal plants and their endophytes can be a potential source for molecules with antioxidant activity [22, 44]. The screening of fungal extracts for free radical scavenging activity via DPPH assay, showed that *C. nigricolor* ethyl acetate extract exhibited the strongest activity followed by the extracts of *Lasiodiplodia theobromae* (isolate 13) and *Colletotrichum* (isolate 19). *C. nigricolor* extracts also exhibited a mediocre level of super oxide scavenging potential. Thus, it is observed that *C. nigricolor* showed significant cytotoxic, anticancer and antioxidant potential.

## Conclusion

The study provides insight into the diversity of endophytic fungal community isolated from *C. roseus* growing in the coastal regions of Mahabalipuram. This is the first report where studies on the diversity of endophytic fungus that inhabit *C. roseus* plant growing in coastal region has been carried out. The data obtained shows that of the 20 endophytic fungal extracts screened for cytotoxic and antioxidant potential, the *C. nigricolor* extract exhibited significant cytotoxic, antioxidant and apoptotic potential.Though the ethyl acetate extract of *C. nigricolor* has been reported for its cytotoxic activity against the HeLa cells previously [45], we have for the first time reported its extracts for bioactivity such as antioxidant, apoptotic and mitochondrial membrane depolarization potential.It is hypothesized that it could harbor metabolites that could serve as promising anticancer and antioxidant agents.

## Additional file

Additional file 1: **Table S1.** Cytotoxicity IC_50_ values of ethyl acetate extracts of *C. nigricolor* grown using different media on HeLa cells. The fungus *C. nigricolor* was grown in the above-mentioned media for 21 days, harvested, extracted using ethyl acetate and evaluated for its cytotoxic activity. Cytotoxicity was determined by the MTT assay using human cervical cancer cell line, HeLa. IC_50_ values are mean ± SD calculated from results obtained from triplicate of two independent experiments (*n* = 6). **Fig. S1** Cytotoxicity of EA and hexane extracts of *C. nigricolor* grown using M-1D medium tested on HEK 293 T (Non cancerous cells). The fungus *C. nigricolor* was grown in the above-mentioned medium as a liquid culture for 21 days, harvested, extracted using ethyl acetate or hexane and evaluated for its cytotoxic activity on HEK 293 T cells using the MTT assay. Percentage of dead cells on treatment with different concentrations of A) EA extract, B) EA mycelial extract C) EA culture filtrate extract D) Hexane culture filtrate extract are represented as bar graphs. Mean ± SD calculated from results obtained from triplicate of two independent experiments (n = 6). (DOCX 107 kb)

## Abbreviations

2,4 DNP: 2,4-Dinitrophenol
Abs: Absorbance
CF: Colonization frequency
DMEM: Dulbecco’s Modified Eagle’s Medium
DMSO: Dimethyl sulphoxide
DPPH: 2,2-diphenyl-1-picrylhydrazyl
EA: Ethyl acetate
EC_50_: Half maximal effective concentration
FBS: Fetal Bovine Serum
FITC: Fluorescein isothiocyanate
h: hour
IC_50_: Half maximal inhibitory concentration
ITS: Internal Transcribed Spacer
MIDA: Modified medium-1 agar
MIDB: Modified medium-1 Broth
mins: minutes
MTT: 3-(4,5-dimethylthiazol-2-yl)-2,5-diphenyltetrazolium bromide
NADH: nicotinamide adenine dinucleotide (NAD) + hydrogen (H)
NtB: Nutrient Broth
OD: Optical density
PDA: Potato dextrose agar
PDB: Potato dextrose broth
PI: Propidium Iodide
PMS: Phenazine methosulfate
SDB: Sabouraud Broth
TBA: Thiobarbituric acid
TCA: Trichloroacetic acid
