# Supplementary material for: Diversity and biological activities of endophytic fungi associated with Catharanthus roseus
Source: BMC Microbiol. 2019 Jan 21;19:22. doi: 10.1186/s12866-019-1386-x (PMC6341747; doi:10.1186/s12866-019-1386-x)
Supplement: Supplementary file 1 — Table S1. Cytotoxicity IC50 values of ethyl acetate extracts of C. nigricolor grown using different media on HeLa cells. The fungus C. nigricolor was grown in the above-mentioned media for 21 days, harvested, extracted using ethyl acetate and evaluated for its cytotoxic activity. Cytotoxicity was determined by the MTT assay using human cervical cancer cell line, HeLa. IC50 values are mean ± SD calculated from results obtained from triplicate of two independent experiments (n = 6). Fig. S1 Cytotoxicity of EA and hexane extracts of C. nigricolor grown using M-1D medium tested on HEK 293 T (Non cancerous cells). The fungus C. nigricolor was grown in the above-mentioned medium as a liquid culture for 21 days, harvested, extracted using ethyl acetate or hexane and evaluated for its cytotoxic activity on HEK 293 T cells using the MTT assay. Percentage of dead cells on treatment with different concentrations of A) EA extract, B) EA mycelial extract C) EA culture filtrate extract D) Hexane culture filtrate extract are represented as bar graphs. Mean ± SD calculated from results obtained from triplicate of two independent experiments (n = 6). (DOCX 107 kb) [file 12866_2019_1386_MOESM1_ESM.docx]

**Additional file 1**

Table S1: Cytotoxicity IC_50_ values of ethyl acetate extracts of *C. nigricolor* grown using different media tested on HeLa cells.

| Extracts of  *C.nigricolo*r  grown in different media | Cytotoxic activity  IC _50_  (µg mL^-1^) |
| --- | --- |
| PDB extract | 38 ± 0.2 |
| SDB extract | 60 ± 0.0 |
| M-1DB extract | 32 ± 0.07 |
| NtB extract | > 100 |

PDB-Potato dextrose broth; (M-1D)B- Modified medium-1 broth; SDB-Sabouraud broth; NtB-Nutrient broth

The fungus *C. nigricolor* was grown in the above-mentioned media for 21 days, harvested, extracted using ethyl acetate and evaluated for its cytotoxic activity. Cytotoxicity was determined by the MTT assay using human cervical cancer cell line, HeLa. IC_50_ values are mean± SD calculated from results obtained from triplicate of two independent experiments (n = 6).

Figure S1 : Cytotoxicity of EA and hexane extracts of *C. nigricolor* grown using M-1D medium tested on HEK 293 T (Non cancerous cells).


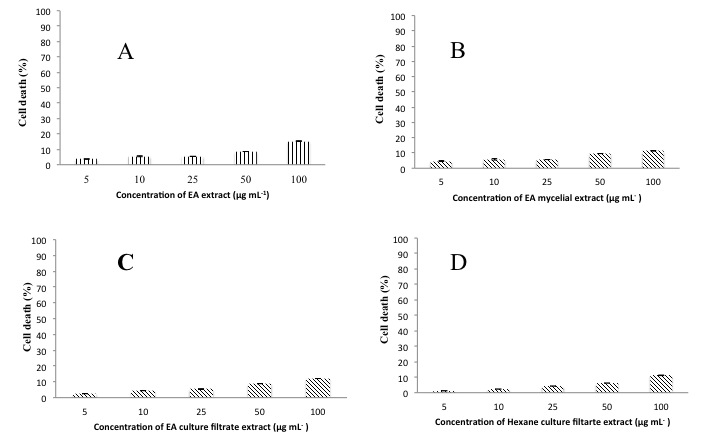


The fungus *C. nigricolor* was grown in the above-mentioned medium as a liquid culture for 21 days, harvested, extracted using ethyl acetate or hexane and evaluated for its cytotoxic activity on HEK 293 T cells using the MTT assay. Percentage of dead cells on treatment with different concentrations of A) EA extract, B) EA mycelial extract C) EA culture filtrate extract D) Hexane culture filtrate extract are represented as bar graphs. Mean± SD calculated from results obtained from triplicate of two independent experiments (n = 6).
